# Zinc improves sexual performance and erectile function by preventing penile oxidative injury and upregulating circulating testosterone in lead-exposed rats

**DOI:** 10.1080/13510002.2023.2225675

**Published:** 2023-06-22

**Authors:** Elizabeth Enohnyket Besong, Tunmise Maryanne Akhigbe, Precious Jesutofunmi Ashonibare, Abimbola Ayoola Oladipo, Jacinta Nkechi Obimma, Moses Agbomhere Hamed, Damilare Hakeem Adeyemi, Roland Eghoghosoa Akhigbe

**Affiliations:** a Department of Physiology, Faculty of Basic Medical Sciences, Ebonyi State University, Abakaliki, Nigeria; b Breeding and Plant Genetics Unit, Department of Agronomy, Osun State University, Osogbo, Osun State, Nigeria; c Reproductive Biology and Toxicology Research Laboratory, Oasis of Grace Hospital, Osogbo, Osun State, Nigeria; d Department of Physiology, Ladoke Akintola University of Technology, Ogbomoso, Oyo State, Nigeria; e Department of Medical Laboratory Science, Afe Babalola University, Ado-Ekiti, Ekiti State, Nigeria; f The Brainwill Laboratory, Osogbo, Osun State, Nigeria; g Department of Physiology, Faculty of Basic Medical Sciences, College of Health Sciences, Osun State University, Osogbo, Osun State, Nigeria

**Keywords:** Erection, heavy metals, infertility, lead, oxidative stress, supplements, testosterone, zinc

## Abstract

**Aim:**

The present study evaluated the effect of lead exposure with and without zinc therapy on male sexual and erectile function.

**Methods:**

Twenty male Wistar rats were randomly assigned into four groups; the control, zinc-treated, lead-exposed, lead + zinc-treated groups. Administrations were per os daily for 28 days.

**Results:**

Zinc co-administration significantly improved absolute and relative penile weights and the latencies and frequencies of mount, intromission, and ejaculation in lead-exposed rats. Also, zinc ameliorated lead-induced reductions in motivation to mate and penile reflex/erection. These findings were accompanied by attenuation of lead-induced suppression of circulating nitric oxide (NO), penile cyclic guanosine monophosphate (cGMP), dopamine, serum luteinizing hormone, follicle-stimulating hormone, and testosterone. In addition, zinc alleviated lead-induced upregulation of penile activities of acetylcholinesterase and xanthine oxidase (XO), and uric acid (UA) and malondialdehyde (MDA) levels. Furthermore, zinc ameliorated the lead-induced decline in penile nuclear factor erythroid 2-related factor 2 (Nrf2) and reduced glutathione (GSH) levels, and catalase, superoxide dismutase (SOD), glutathione peroxidase (GPx), and glutathione-S-transferase (GST) activities.

**Conclusion:**

This study revealed that co-administration of zinc improves lead-induced sexual and erectile dysfunction by suppressing XO/UA-driven oxidative stress and upregulating testosterone via Nrf2-mediated signaling.

## Introduction

Heavy metals are various unrelated elements with a specific density greater than 5 g/cm^3^ [1]. They are greatly abundant in the environment, with naturally occurring toxicity and also due to human activities [2]; hence, they are considered common environmental pollutants [3]. These molecules are typically non-steroidal organic chemicals commonly released into the atmosphere via human physical activities like industrial activities, agricultural spraying, urban waste, and/or consumer products [4]. They exert toxic effects on various organs [5–8], including the testis and spermatozoa [9,10]. Heavy metals; through their actions on estrogen receptors, act as endocrine-disrupting chemicals by altering hormone concentrations, affecting the synthesis or metabolism of hormones, or modifying the hormonal actions [11]. They may also exert endocrine disruption by mimicking or inhibiting their primary targets, especially oestrogens, progestins, and androgens [11,12]. These heavy metals include aluminium, cadmium, mercury, copper, and lead.

Lead has a very great impact on the reproductive organs in males, and this may involve several pathways in lead-induced male reproductive dysfunction [13–15]. It has been reported that certain reproductive parameters are affected by exposure to low-to-moderate levels of environmental lead [16]. Lead decreases male fertility by reducing the quality of spermatozoa, although the blood-testis barrier protects testicular tissue from being directly exposed to increased lead concentrations in the blood [15]. It has also been reported that the hypothalamic-pituitary-testicular axis may be adversely affected due to occupational and environmental exposure to lead, thus impairing testicular steroidogenesis and spermatogenesis. Lead poisoning also affects the process of spermatogenesis and the function of spermatozoa; excess reactive oxygen species (ROS) generated as a result of exposure to lead reduces the viability and motility of the spermatozoa, promotes DNA fragmentation, and impairs chemotaxis for spermatozoa–oocyte fusion, which could aid a decline in fertilization [17]. A blood lead level greater than 40 µg/dl has been associated with the impairment of male reproductive functions by reducing the volume, count, and density of spermatozoa; also altering the morphology and motility of the spermatozoa [18]. The total spermatozoa count is inversely proportional to the blood-lead levels, and as the concentrations of lead in semen increase, there is a decrease in the total spermatozoa count, ejaculate volume, and serum testosterone [19,20]. In addition, lead markedly alters testicular architecture [21]. The study of Elsheikh et al. [21] revealed that lead exposure shrank the seminiferous tubules with a complete cessation of spermatogenesis. Lead-induced toxicity has been shown to be via the induction of oxidative stress [22,23]. This is mediated by the generation of ROS that exceeds the cellular antioxidant defense capacity [24,25]. Lead exposure may also decrease the activities of antioxidants [26] and/or the concentrations of antioxidants by enhancing nitric oxide (NO) production via the upregulation of NO synthase [27]. However, to date, there is a paucity of data on the effect of lead and the associated mechanisms of action on male sexual and erectile function.

Zinc is an essential trace element required for many physiological processes, and its deficiency has been associated with various pathological conditions [28]. Zinc serves as a co-factor for 300 enzymes and 2000 transcription factors, and also mediates several cellular signaling pathways [29,30]. Zinc stabilizes membranes, inhibits NADPH-oxidase, and induces metallothionein synthesis, thus contributing to the proper functioning of the antioxidant defense system and protecting cells against oxidative damage [31,32]. Zinc also provides structural stability to cell membrane functions, exerts anti-inflammatory activities, and plays a vital role in the regulation of gene expression [33,34]. Zinc has been shown to maintain optimal testosterone levels [35,36], which in turn may be beneficial in suppressing inflammation [37] and preserving penile endothelial function; hence, promoting erectile function and male sexual activity [38]. In our recent study [35], we demonstrated that zinc improved sexual and erectile function in highly active antiretroviral-induced sexual dysfunction by maintaining penile redox balance and upregulating erectogenic enzymes. Nevertheless, the protective function of zinc in lead-induced sexual and erectile dysfunction is yet to be reported.

Since lead has been reported to reduce circulatory testosterone levels, a major mediator of optimal sexual and erectile function, via an oxidative stress-mediated pathway, and zinc possesses antioxidant properties with a potential to improve erectogenic enzymes, we thus hypothesized that zinc will restore lead-induced impairment of male sexual performance and erectile function. To test this hypothesis, we exposed male Wistar rats to lead with or without zinc supplementation by gavage for 28 days. In addition, the involvement of oxidative stress and circulating testosterone levels in lead-induced impairment of male sexual performance and erectile function was investigated.

## Materials and methods

### Animals and treatment

This study conforms to the guidelines of the National Institute of Health Guide for the Care and Use of Laboratory Animals and is reported in line with the ARRIVE guidelines. The Institutional Ethical Review Committee approved this study. Minimal numbers of rats were used, and they were subjected to humane care. Littermate male Wistar rats of about 10 weeks old with similar weights were used. The animals were kept in clean plastic cages (n = 5/cage) in a well-ventilated room under the natural conditions of the light/dark cycle. Animals fed on rat chow and drank clean water at liberty. The rats were grouped at random into four groups (n = 5/group) after 14 days of acclimatization.

The control group was vehicle-treated daily with 0.5 ml of distilled water, the zinc-treated group was given zinc (3 mg/kg/day of elemental zinc), the lead-treated group was given lead acetate (20 mg/kg), and lead + zinc-treated rats were given lead acetate and zinc as in the lead-treated and zinc-treated groups. Zinc was administered thirty minutes before lead exposure in the fourth group. Treatment was by gavage for 28 days. The dose of zinc administered is equivalent to about 30 mg/day for humans, which is about 11 mg (the recommended dietary allowance) and the tolerable upper intake level of 40 mg [39], while the dose and route of lead administration [40] and duration of exposure [41] are as previously reported.

### Assessment of sexual and erection function

The penile reflex was elicited and scored as previously reported [38,42]. Each male rat was minimally restrained in a supine position, and then the preputial sheath was retracted behind the glans penis and maintained in this position for about 15 min to elicit genital reflexes. The sum of erection (E), long flips (LP), and quick flips (QF) was used to calculate total penile reflexes (TPR), an index of penile erection and function, i.e. [TPR = E + QF + LF].

Sexual performance was assessed 24 h after the last dose of zinc and lead. Animals were put in transparent plastic cages with a wire-mesh top (n = a rat/cage) and paired with an oestrous-induced female rat (male: female, 1: 1) in a quiet room under dim light. Oestrus was induced by subcutaneous administration of progesterone (0.5 mg/100 g body weight) and oestradiol benzoate (10 mg/100 g body weight) at 4 and 48 h, respectively, before pairing [43,44] and confirmed by visual assessment of the vaginal and vaginal smear [43]. Only receptive female rats were selected for the study. Sexual behavior was monitored for 30 min using a camcorder.

Motivation to mate (an index of libido) was assessed as earlier reported [45] (SI Table 1). Latencies and frequencies of mount, intromission, and ejaculation, as well as post-ejaculatory interval, were determined as indices of sexual function as previously documented [42,46] (SI Table 2).

### Sacrifice and tissue collection

Rats were weighed before and after the study and recorded as their initial and final body weights, respectively. The difference in these weights was obtained and recorded as the change in body weight. At the end of the experimental period, animals were euthanized with ketamine (40 mg/kg *i.p.*) and xylazine (4 mg/kg *i.p.*). Blood samples were collected through the retro-orbital sinus into appropriate sample bottles and centrifuged at 3000 rpm for 5 min to obtain the plasma, which was stored frozen until it was needed for assays. The penis was harvested, separated from adhering structures, blotted, and weighed immediately to obtain the absolute and relative penile weights. The relative penile weight was obtained as the absolute penile weight/final body weight × 100%. The epidermis was removed and the tunica albuginea opened, then the corpus cavernosum was dissected out. About 100 mg of the cavernosal tissue was obtained and homogenized in cold phosphate-buffered solution (1: 5) using a glass homogenizer and centrifuged at 10,000 g for 15 min at 4°C to obtain the supernatant, which was stored at −20°C for biochemical assay.

### Evaluation of lead concentration

The levels of lead in the blood and testes were assayed as earlier reported by Zhang et al. [47]. Approximately 0.6 mL of 30% H_2_O_2_ and 2.4 mL of 65% HNO_3_ were mixed, then 500 μL blood or 100 mg testicular tissue was added and left at room temperature for more than 12 h to allow digestion of the solution. The samples were heated at 150°C until the solution was clear and transparent. The volume of the solution was made up to 2 mL by adding 0.5% HNO_3_. The same procedure was repeated with blank reagents without blood and testicular tissue samples. All sample supernatants were used to detect the lead concentrations. Samples were measured using a flame atomic absorption spectrophotometer at 283.3 nm.

### Assessment of NO/cGMP signaling and erectogenic enzymes

Penile nitric oxide (NO) was assayed based on the Griess reaction using a standard ELISA kit (Biovision Research Products, USA), while the principle of protein-binding using a standard ELISA kit (Elabscience Biotechnology Inc., USA) was used to determine penile cyclic guanosine monophosphate (cGMP).

The activity of penile acetylcholinesterase (AchE), an enzyme that hydrolyzes acetylcholine (a neurotransmitter involved in arousal), was used as an index of sexual arousal and assayed using an ELISA kit (Elabscience Biotechnology Inc., USA) following the manufacturer's guidelines. Dopamine concentration was also used as an index of sexual motivation and arousal and assayed using an ELISA kit (Abnova, UK) following the manufacturer's guidelines.

### Assessment of male reproductive hormones

Circulating levels of follicle-stimulating hormone (FSH), luteinizing hormone (LH), and testosterone were determined by spectrophotometry using ELISA kits (Monobind Inc., USA) following the manufacturer's guidelines.

### Assessment of XO/UA signaling

Penile xanthine oxidase (XO) activities (Fortress Diagnostic, Antrim, UK), and uric acid (UA) concentrations (Precision Kit, India) were analyzed by colorimetric methods using standard assay kits. XO assay was based on the color intensity generated from the oxidation of xanthine to H_2_O_2_ by XO [39], while UA assay was based on the conversion of UA by uricase into allantoin, carbon dioxide, and hydrogen peroxide that gives a color indicator reaction that is measured at a wavelength of about 520 nm with the action of hydrogen peroxidase [48,49].

### Assessment of oxidative stress markers and activities of enzymatic antioxidants

The penile concentrations of malondialdehyde (MDA) and reduced glutathione (GSH) were used as indices of oxidative stress. MDA was assayed by colorimetric methods based on the thiobarbituric acid method by quantifying the thiobarbituric acid reactive substance (TBARS) produced during lipid peroxidation [50] using standard laboratory kits (Oxford Biomedical Research, Inc., Oxford, USA). GSH concentration was assayed by the colorimetric method as reported by Beutler et al. [51]. The penile activities of catalase [52], superoxide dismutase (SOD) [7,53], glutathione peroxidase (GPx) [54], and glutathione-S-transferase (GST) [55] were assayed using established protocols as previously reported. The penile concentration of nuclear factor erythroid 2-related factor 2 (Nrf2) was determined using an ELISA kit (Elabscience Biotechnology Inc., USA) following the manufacturer's guidelines.

### Statistical analysis

The mean± standard error of the mean (SEM) was used to present and express the data. Data analysis was carried out utilizing GraphPad Prism (Prism 7 for Windows, Version 7.00). One-way analysis of variance (ANOVA) was used to determine the significance of the difference between the groups, and then Tukey's post hoc test was used for pair-wise comparisons. *P* values < 0.05 were regarded as significant.

## Results

### Lead concentrations in the blood and corpus cavernosum

Lead significantly increased the levels of lead in the blood and cavernosal tissue. Co-administration of zinc attenuated lead-induced rise in blood and cavernosal lead levels (Table 1).

**Table 1.** Blood and corpus cavernosal concentrations of lead after the 28-day experimental period male Wistar rats.

	Control	Zinc-treated	Lead-treated	Lead+Zinc-treated
Lead concentration				
Blood (µg/dL)	0.910±0.010	0.820±0.020	10.700±0.110*#	6.77±0.100*#∼
Corpus cavernosum (µg/g)	0.032±0.003	0.024±0.002	0.214±0.015*#	0.110±0.008*#∼

Values are mean ± SEM of 5 replicates. Data were analyzed by one-way ANOVA followed by Tukey’s post hoc test. *P < 0.05 vs. control, #*P* < 0.05 vs. zinc-treated, ∼*P* < 0.05 vs. lead-treated.

### Body and penile weight

Rats with similar weights were used for the study; hence, there is no tangible difference in the initial body weight across the groups. The final body weight and body weight change were significantly lower in lead-exposed rats, which were robustly ameliorated by zinc treatment. Consistently, the absolute and relative penile weights, indices of penile homeostasis, and penile neural and DNA integrity were markedly lower compared to the control group. These changes were dampened by zinc co-treatment in animals that received lead and zinc treatment when compared with lead-treated rats (Table 2). The results indicate that lead exposure disrupted cellular metabolism and growth, and also impaired penile integrity, which could be improved by zinc therapy.

**Table 2.** The effect of zinc on body weight and penile weight in lead-treated male Wistar rats.

	Control	Zinc-treated	Lead-treated	Lead+Zinc-treated
Initial body weight (g)	141.2±0.73	141.6±0.96	140.9±0.91	141.5±0.71
Final body weight (g)	171.4±1.03	172.8±1.11	156.4±1.74*#	171.8±0.86∼
Change in body weight (g)	30.20±1.46	31.60±1.72	15.20±1.82*#	30.60±1.40∼
Absolute penile weight (g)	1.16±0.03	1.18±0.01	0.74±0.03*#	1.03±0.07*#∼
Relative penile weight (%)	0.61±0.01	0.62±0.02	0.42±0.02*#	0.53±0.04*#∼

Values are mean ± SEM of 5 replicates. Data were analyzed by one-way ANOVA followed by Tukey’s post hoc test. *P < 0.05 vs. control, #*P* < 0.05 vs. zinc-treated, ∼*P* < 0.05 vs. lead-treated.

### Sexual and erection function

**Figure 1.**
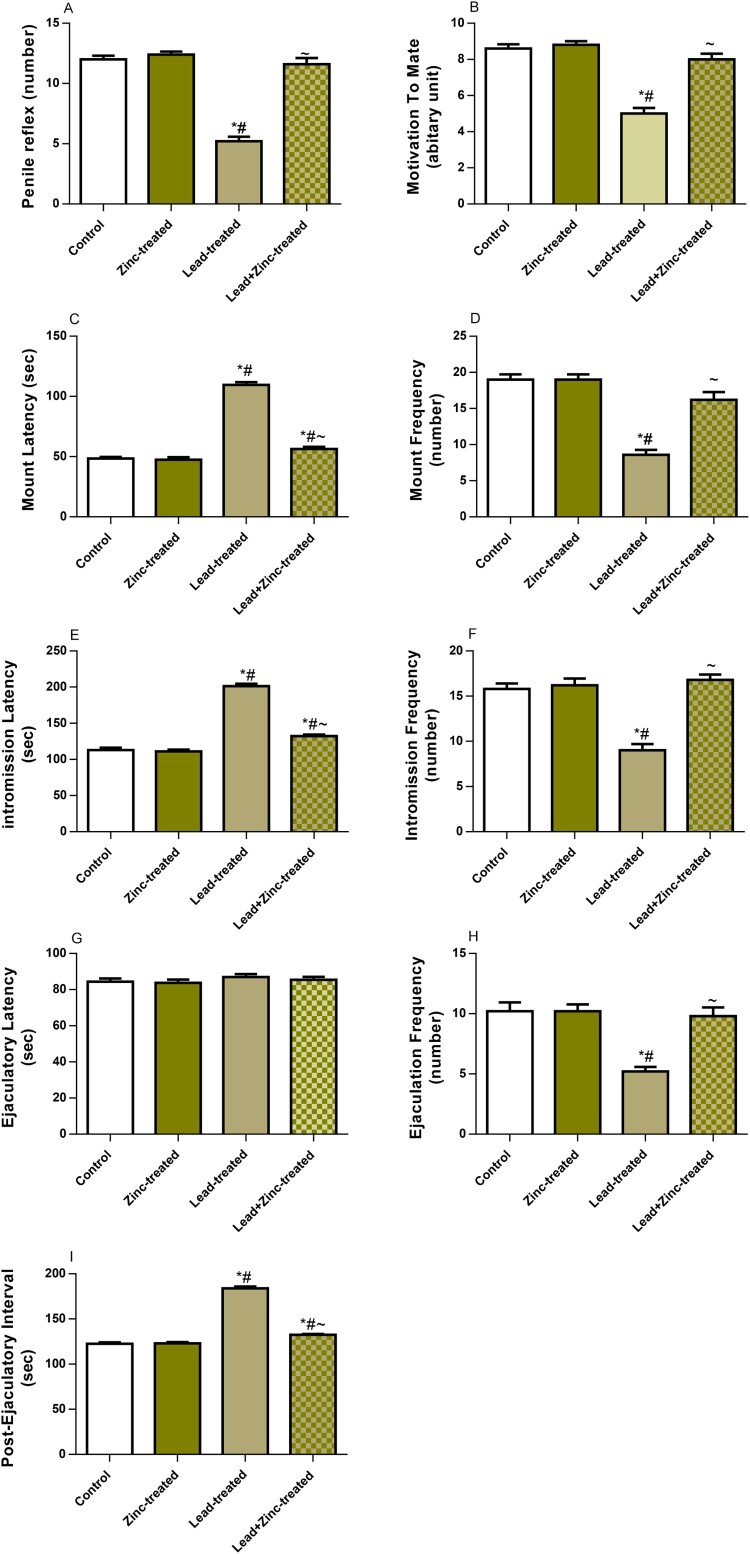
The effect of zinc on penile reflex (A), motivation to mate (B), mount latency (C), mount frequency (D), intromission latency (E), intromission frequency (F), ejaculation latency (G), ejaculation frequency (H), and post-ejaculatory interval (I) in lead-treated male Wistar rats. Values are mean ± SEM of 5 replicates. Data were analyzed by one-way ANOVA followed by Tukey's post hoc test. *P < 0.05 vs. control, #*P* < 0.05 vs. zinc-treated, ∼*P* < 0.05 vs. lead-treated.

In this study, we found that lead exposure significantly reduced penile reflex and motivation to mate. The reductions in penile reflex and motivation to mate were significantly alleviated by zinc supplementation in animals that received lead and zinc treatment when compared with lead-treated rats (Figure 1 A, B). While lead exposure significantly increased the latencies of mount and intromission as well as post-ejaculatory interval, it reduced the frequencies of mount, intromission, and ejaculation. These alterations were ameliorated by zinc therapy in animals that received lead and zinc treatment when compared with lead-treated rats (Figure 1 C-I). This suggests that lead exposure impaired sexual urge and vigor, and penile erection, which may be improved by zinc supplementation.

### NO/cGMP signaling and erectogenic enzymes

**Figure 2.**
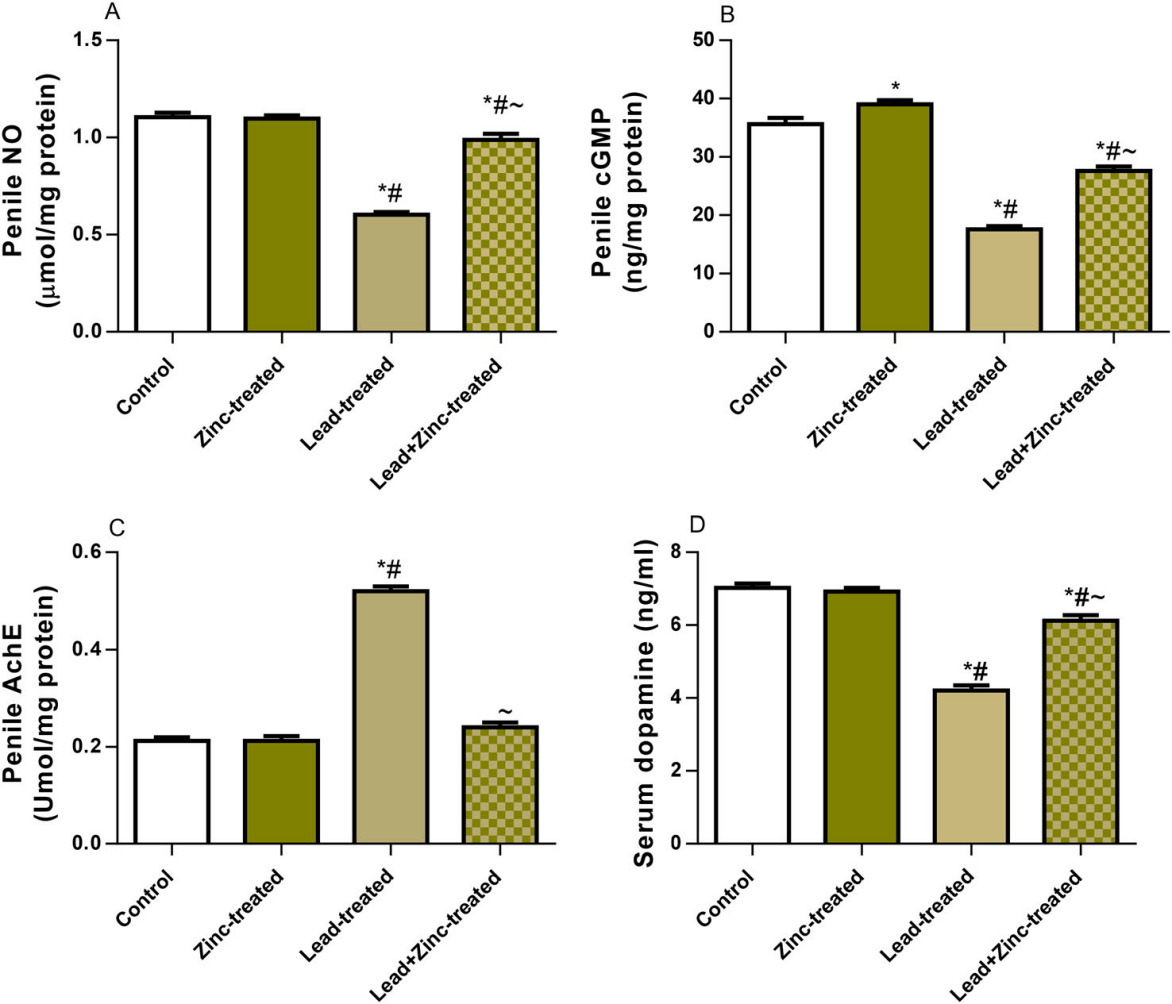
The effect of zinc on serum nitric oxide, NO (A), penile cGMP (B), penile acetyl cholinesterase, AchE (C), and serum dopamine (D) in lead-treated male Wistar rats. Values are mean ± SEM of 5 replicates. Data were analyzed by one-way ANOVA followed by Tukey's post hoc test. *P < 0.05 vs. control, #*P* < 0.05 vs. zinc-treated, ∼*P* < 0.05 vs. lead-treated.

To explore the mechanism of zinc therapy to alleviate lead-induced sexual dysfunction, NO/cGMP, AchE, and dopamine were determined. Circulating NO, penile cGMP, and dopamine were significantly reduced in the lead-exposed group as compared to the vehicle-treated control. In contrast, zinc supplementation in the lead-exposed rats led to a significant increase in NO, penile cGMP, and dopamine when compared to the lead-exposed group. Furthermore, lead exposure significantly increased AchE activity, which was ameliorated by zinc treatment in animals that received lead and zinc treatment when compared with lead-treated rats (Figure 2). This suggests that lead exposure inhibits erectogenic neurotransmitters, but zinc treatment could improve these mediators altered by lead exposure.

### Circulating male reproductive hormones

**Figure 3.**
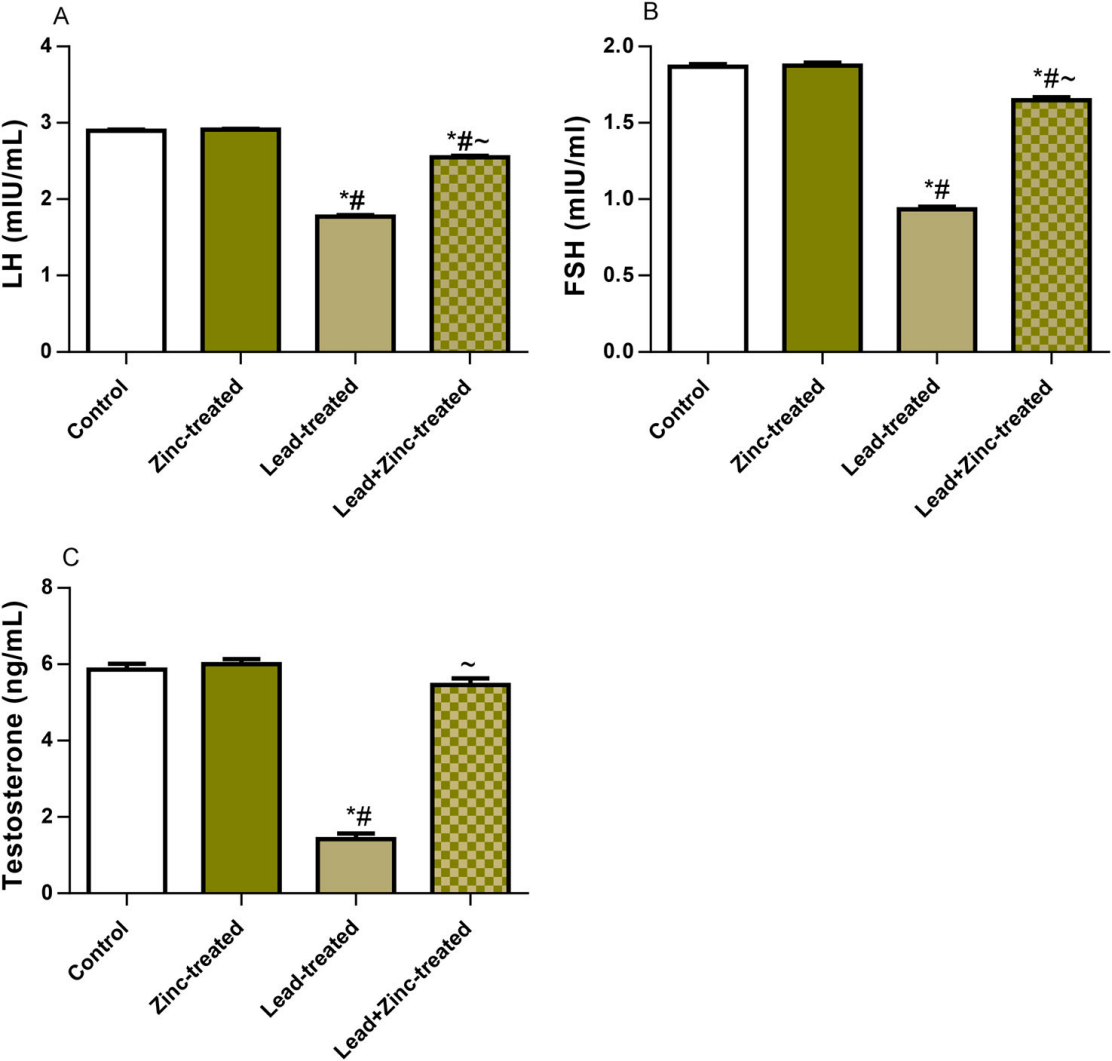
The effect of zinc on luteinizing hormone, LH (A), follicle-stimulating hormone, FSH (B), and testosterone (C) in lead-treated male Wistar rats. Values are mean ± SEM of 5 replicates. Data were analyzed by one-way ANOVA followed by Tukey's post hoc test. *P < 0.05 vs. control, #*P* < 0.05 vs. zinc-treated, ∼*P* < 0.05 vs. lead-treated.

The circulating levels of LH, FSH, and testosterone are shown in Figure 3. Compared to the vehicle-treated control rats, the lead-treated rats showed a marked decline in serum levels of LH, FSH, and testosterone. The observed lead-induced alterations in the levels of these hormones were ameliorated by zinc treatment in animals that received lead and zinc treatment when compared with lead-treated rats. These findings suggest that zinc supplementation ameliorates lead-induced suppression of the pituitary-testicular axis and promotes testosterone synthesis and release.

### Oxidative stress markers and the activities of enzymatic antioxidants

According to the available literature, oxidative stress is an integral process in lead-induced injury, and increasing attention has been paid to triggers and blockers of oxidative stress over the years. However, the nexus between lead exposure and oxidative stress in sexual and erectile dysfunction and the possible role of zinc are understudied. To further evaluate the mechanisms associated with lead-induced sexual dysfunction and the possible role of zinc, we investigated the change in the penile redox state.

**Figure 4.**
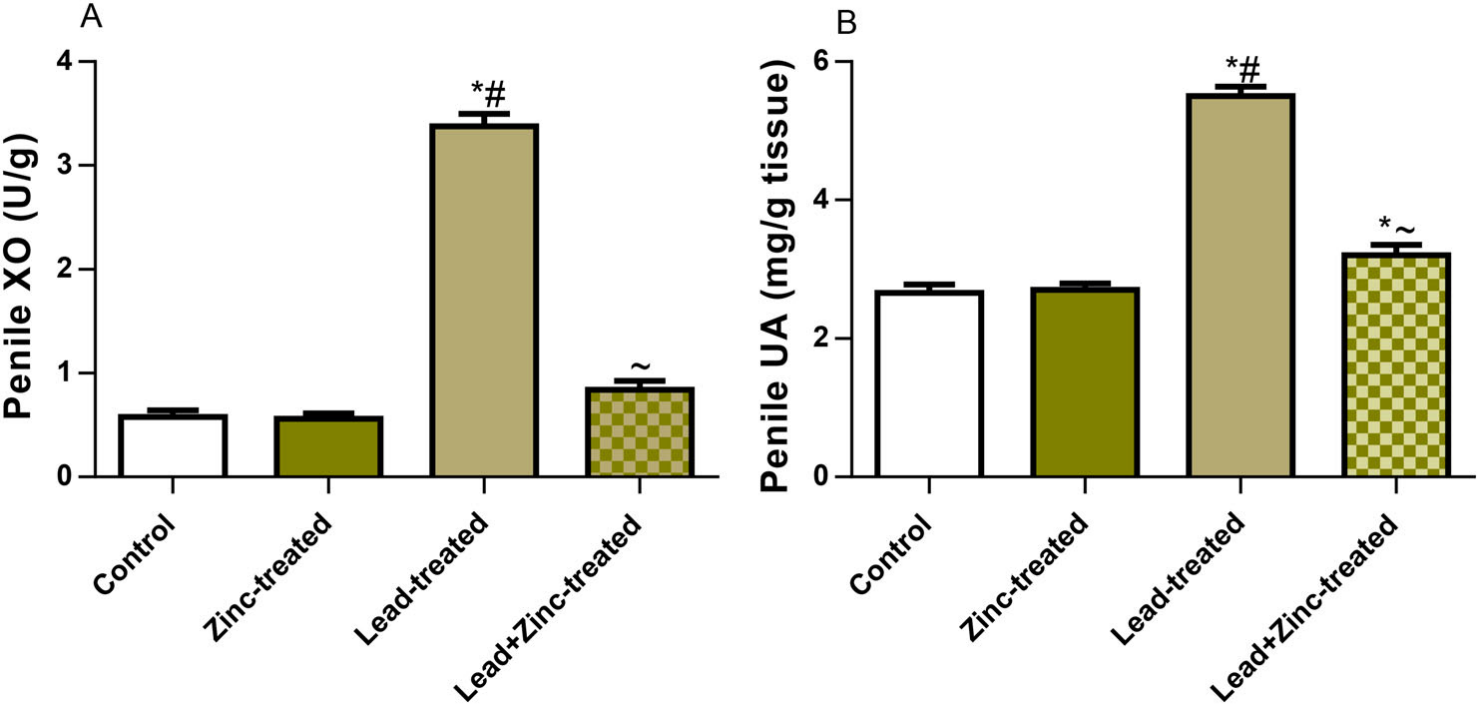
The effect of zinc on penile xanthine oxidase, XO (A) and uric acid, UA (B) in lead-treated male Wistar rats. Values are mean ± SEM of 5 replicates. Data were analyzed by one-way ANOVA followed by Tukey's post hoc test. *P < 0.05 vs. control, #*P* < 0.05 vs. zinc-treated, ∼*P* < 0.05 vs. lead-treated.

**Figure 5.**
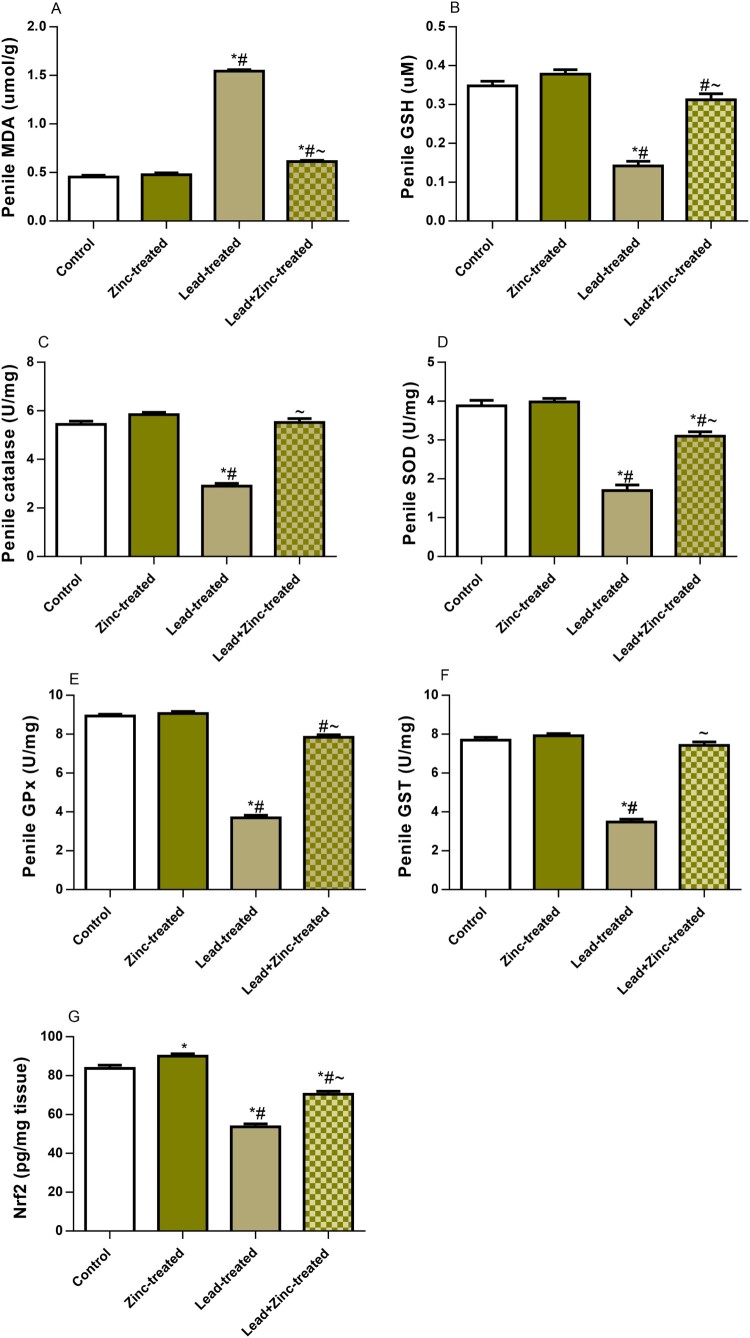
The effect of zinc on penile contents of malondialdehyde, MDA (A) and reduced glutathione, GSH (B), activities of catalase (C), superoxide dismutase (D), glutathione peroxidase, GPx (E) and glutathione-S-transferase, GST (F), and nuclear factor erythroid 2-related factor 2, Nrf2 (G) concentration in lead-treated male Wistar rats. Values are mean ± SEM of 5 replicates. Data were analyzed by one-way ANOVA followed by Tukey's post hoc test. *P < 0.05 vs. control, #*P* < 0.05 vs. zinc-treated, ∼*P* < 0.05 vs. lead-treated.

As shown in Figure 4, penile XO activity and UA levels, which are triggers of oxidative stress, were markedly increased in the lead-exposed group compared to the control group. Lead-induced increases in penile XO activity and UA levels were significantly attenuated by zinc supplementation in animals that received lead and zinc treatment when compared with lead-treated rats. More so, MDA, a product of oxidant-triggered lipid peroxidation and a marker of lipid peroxidation, was significantly increased in lead-exposed rats when compared to the control group. This was nullified by zinc treatment in lead-exposed rats. In addition, lead exposure significantly reduced GSH levels, which were abrogated by zinc co-treatment in animals that received lead and zinc treatment when compared with lead-treated rats. Furthermore, lead markedly reduced the penile activities of catalase, SOD, GPx, and GST compared to the vehicle-treated control. The decline observed in the activities of these enzymatic antioxidants was reversed by co-treatment with zinc in animals that received lead and zinc treatment when compared with lead-treated rats. Additionally, Nrf2, a mediator and regulator of cellular defense against oxidative stress, was observed to be markedly reduced in lead-exposed rats. The lead-induced decline in penile Nrf2 was ameliorated by zinc supplementation in lead-exposed rats (Figure 5). These results show that zinc supplementation improved lead-induced penile oxidative injury by suppressing XO/UA signaling and upregulating Nrf2 expression.

## Discussion

Sexual and erectile dysfunction is a complex and discrete pathological process that is primarily differentiated into two parts: impairment of sexual urge and vigor, and erectile dysfunction. Although this may be psychogenic, neurogenic, or arteriogenic [56]; it is hormone-dependent in most cases [37]. Environmental toxicants, including heavy metals such as lead, have been reported to reduce circulating testosterone, but their impact on sexual performance and erectile function is under-reported. Therefore, exploring the effect and mechanisms of action of heavy metal exposure on sexual performance and erectile function is pertinent. Also, the development of effective preventive measures against sexual and erectile dysfunction resulting from exposure to heavy metals is important for improving male reproductive health. In this study, it was demonstrated for the first time that lead exposure may result in sexual and erectile dysfunction and that zinc supplementation may improve lead-induced sexual and erectile dysfunction. Mechanistically, the pathological process borders on the activation of oxidative stress and suppression of circulating testosterone. The present data not only provide convincing evidence of the effect of lead on sexual and penile erection but also shine a light on the therapeutic potentials of zinc in improving sexual performance and erectile function via a testosterone-dependent pathway linked with the repression of oxidative stress.

Although little or no attention is given to penile homeostasis in male sexual activity, our previous study revealed that impaired penile reflexes were associated with penile dyshomeostasis and reduced penile neural and DNA integrity [57]. However, several studies have implicated impaired penile reflexes in incidents of sexual and erectile dysfunction [38,58]. Notably, we noted that lead exposure-mediated reductions in penile weight are closely associated with impaired penile reflexes. This finding implies that the effect of lead exposure on penile homeostasis is a crucial issue and deserves further attention. In addition, penile reflexes play an important role in sexual drive and may be stimulated by audiovisual or tactile stimuli [56]. Therefore, the reduced penile reflexes caused by lead exposure may account for the reduced motivation to mate, which provides a novel direction for lead exposure-induced low sex drive.

The present study also demonstrated that lead exposure markedly increased the latencies of mount and intromission, which are indices of sexual motivation and are inversely proportional to it [38,59]. The delayed mount and intromission latencies observed in the present study following lead exposure hint that exposure to lead suppresses sexual motivation and arousal. These variables (mount and intromission latencies) are also predictors of sexual appetitive behavior [38]; hence, lead-induced increase in mount and intromission latencies is a reflection of the sexual reluctance of the male rats towards the receptive female. More so, lead exposure reduced the frequencies of mount, intromission, and ejaculations, which are indices of libido and sexual vigor [60]. The index of potency and rate of restoration from exhaustion after the first series of sexual acts is the post-ejaculatory interval [61]. Lead-induced impaired motivation to mate was characterized by a prolonged post-ejaculatory interval, which shows that lead-exposed rats recovered slowly and delayed initiating another round of sexual activity, thus, demonstrating the ability of lead to impair sexual vigor.

Non-androgenic, non-cholinergic-dependent NO-mediated relaxation of the cavernous smooth muscle plays a principal role in penile erection and adequate penetration. Besides its direct vasodilator effect, NO activates guanylyl cyclase through the upregulation of cGMP [62,63]. Therefore, maintenance of optimal levels of cGMP is required for attaining and sustaining penile rigidity. Cholinergic-dependent penile erection is mediated by acetylcholine, a neurotransmitter that is hydrolyzed and inactivated by AchE [63]. Although phosphodiesterase-5 was not evaluated in the present study, the observed lead-induced low cGMP may be due to possible increased PDE-5 activity since it is known to degrade cGMP to inactive GMP [64]. The observed low circulatory NO in lead-exposed animals may also, at least in part, contribute to the reduced cGMP due to impaired NO-dependent conversion of guanosine triphosphate to cGMP. Lead-induced upregulation of AchE may have promoted acetylcholine hydrolysis and inactivation, thus preventing cholinergic-dependent penile erection. AchE-mediated depletion of acetylcholine also impairs NO production [65], thus leading to endothelial dysfunction and poor penile erection. Lead-induced downregulation of NO/cGMP and AchE upsurge reveals that lead-induced sexual and erectile dysfunction involves multiple mechanistic pathways. It is plausible to infer that lead exposure up-regulated AchE that breaks down acetylcholine, resulting in reduced acetylcholine concentration. Since acetylcholine promotes NO synthesis, lead-driven suppression of acetylcholine represses NO production and endothelial function, thus impairs erectile function.

The induction of oxidative stress may contribute to the upregulation of AchE activity observed in lead-exposed rats, thus negatively modifying the cholinergic inflammatory regulatory role of acetylcholine and the consequent release of pro-inflammatory cytokines and free radicals [66,67]. This study provides the first evidence that lead-induced oxidative stress is XO/UA-mediated. Lead exposure increased XO and UA generation, which in turn promoted NOS uncoupling, superoxide generation, and reduced NO bioavailability [68], leading to oxidative stress [69]. This is further substantiated by the lead-induced rise in penile MDA and decline in GSH and Nrf2, as well as the downregulation of catalase, SOD, GPx, and GST activities. This confirms the findings of Zhang et al. [47] that lead-induced reproductive toxicity is mediated by oxidative stress. Although lead may induce direct toxicity in the penile tissue, thus depressing penile metabolism and homeostasis [70], it is likely that lead-induced penile oxidative injury also plays a role in the observed reduction in penile weight. Also, lead-induced oxidative stress may activate redox-sensitive signaling involving cytokines, kinases, and transcription factors that regulate the expression of genes and modify the extracellular matrix, proteins influencing vasoconstriction, and inflammatory processes, resulting in destructive changes in cellular components, including lipids, protein and DNA [42], ultimately leading to smooth muscle degeneration and vascular endothelial injury, thereby inducing arteriogenic erectile dysfunction [71].

Fascinatingly, the lead-induced oxidative penile injury may also be testosterone-mediated. Testosterone exerts dimorphism in the regulation of inflammation [37]. In males, optimal levels of testosterone exert anti-inflammatory activity by inhibiting the upregulation of pro-inflammatory cytokines and promoting the expression and activities of anti-inflammatory cytokines [72], thus preventing inflammation-induced oxidative stress [68,73]. The observed decline in testosterone in lead-exposed rats aligns with and forms an extension of previous human and animal studies [74,75] that observed a decline in circulating testosterone levels following lead exposure. The present findings suggest that lead-induced suppression of circulating testosterone promotes the upregulation of pro-inflammatory cytokines and activation of oxidative stress cascades, resulting in endothelial dysfunction and reduced erectile function. Also, since it has been established that testosterone enhances sexual interest, frequency of sexual acts and erections [76] and pelvic thrusting during copulation [77], the lead-induced decline in testosterone levels may inhibit these testosterone-dependent events. Furthermore, optimal levels of testosterone are essential for the synthesis and release of dopamine, another erectogenic neurotransmitter that facilitates sexual motivation and penile erection [78]. The reduced testosterone level causes a reduction in dopamine synthesis and release, impairment of dopamine-mediated activation of D2 receptors to stimulate NO synthase in the cell bodies of the paraventricular nucleus, and inefficient sexual motivation, urogenital reflexes, and penile erection [62]. The observed cascade of events unfolds striking evidence to show that lead-induced sexual and erectile dysfunction is mediated by complex pathophysiological mechanisms.

Another captivating finding of recent studies is the effect of zinc therapy on lead-induced sexual and erectile dysfunction. Our present finding that zinc up-regulates Nrf2 and the glutathione-dependent antioxidant defense system is in keeping with previous findings [79–81] that demonstrated that zinc promotes Nrf2 expression and function through the activation of Akt-mediated inhibition of the Nrf2 nuclear exporter, Fyn, resulting in the upregulation of the transcription of the catalytic subunit (heavy chain) of glutamate-cysteine ligase (GCLC) and consequent increase in the concentrations of GSH. The observed upregulation of Nrf2-signaling and increased GSH content possibly mediates zinc-induced suppression of XO/UA-driven oxidative stress, thus improving penile homeostasis and DNA integrity, endothelial function, and penile erection. Repression of oxidative stress by zinc also possibly inhibited NO depletion and inactivated AchE, thus enhancing NO/cGMP signaling and cholinergic-dependent penile erection.

More so, our findings that zinc therapy maintains optimal circulating testosterone levels agree with a previous finding that reported a similar effect [36]. Zinc-driven maintenance of optimal testosterone levels may contribute to the stultification of lead-induced inflammation and the improvement of penile erection [37]. It is also safe to infer that the enhanced testosterone concentration associated with zinc supplementation mediates zinc-induced improvement of sexual and erectile function in lead-exposed rats by preserving penile endothelial function and promoting sexual interest, frequency of sexual acts, penile erections [76] and pelvic thrusting during copulation [77].

## Conclusions

The present study revealed that XO/UA-mediated oxidative stress and suppression of testosterone is associated with lead-induced sexual and erectile dysfunction. Co-administration of zinc prevented lead-induced sexual performance and penile erection by activating Nrf2-dependent signaling. This provides a novel mechanistic understanding of the possible role(s) of zinc therapy in lead-induced sexual and erectile dysfunction.

## Declarations

## Supplementary Material

Supplemental Material

## Data Availability

Data will be made available upon reasonable request from the corresponding author.

## Abbreviations

**Abbreviations:** AchE: Acetylcholinesterase; cGMP: Cyclic guanosine monophosphate; DNA: Deoxyribonucleic acid; E: Erection; FSH: Follicle-stimulating hormone; GPx: Glutathione peroxidase; GSH: Reduced glutathione; GST: Glutathione-S-transferase; LH: Luteinizing hormone; LP: Long flips; MDA: Malondialdehyde; NADPH: Nicotinamide adenine dinucleotide phosphate; NO: Nitric oxide; Nrf2: Nuclear factor erythroid 2-related factor 2; QF: Quick flips; ROS: Reactive oxygen species; SOD: Superoxide dismutase; TBARS: Thiobarbituric acid reactive substance; TPR: Total penile reflexes; UA: Uric acid; XO: Xanthine oxidase
