# Supplementary material for: Zinc improves sexual performance and erectile function by preventing penile oxidative injury and upregulating circulating testosterone in lead-exposed rats
Source: Redox Rep. 2023 Jun 22;28(1):2225675. doi: 10.1080/13510002.2023.2225675 (PMC10291914; doi:10.1080/13510002.2023.2225675)
Supplement: Supplemental Material [file YRER_A_2225675_SM8198.doc]

SI Table 1: Assessment of motivation to mate

| **Score** | **Activity observed** |
| --- | --- |
| 0 | no sexual activity |
| 1 | no interaction, rears, and climbs on the chamber |
| 2 | sniffs the female animal |
| 3 | self-exploratory behaviour such as grooming and sniffing of genitals |
| 4 | grooms female counterpart anywhere |
| 5 | rears and climbs sexually |
| 6 | pursues and sniffs the female animal |
| 7 | tries to mount but easily discouraged |
| 8 | mounts with an integrated deliberate manner and not easily discouraged |
| 9 | reflex and almost involuntary mount |

SI Table 2: Assessment of motivation to mate

| **Score** | **Activity observed** |
| --- | --- |
| Mount latency | the time lag between the introduction of the female and the first mount by the male |
| Intromission latency | the time interval between the introduction of the female counterpart and the intromission (penetration) by the male |
| Ejaculation latency | the time interval between the first intromission and ejaculation |
| Mount frequency | the number of mounts from the time of introduction of the female counterpart until ejaculation |
| Intromission frequency | the number of intromissions from the time of introduction of  the female animal until ejaculation |
| Ejaculation frequency | the number of ejaculations from the time of the introduction of the female to the male within 30 min |
| Post-ejaculatory interval | the time interval between ejaculation and the next intromission |
